# Winter bait stations as a multispecies survey tool

**DOI:** 10.1002/ece3.3158

**Published:** 2017-07-27

**Authors:** Lacy Robinson, Samuel A. Cushman, Michael K. Lucid

**Affiliations:** ^1^ Idaho Department of Fish and Game Coeur d'Alene ID USA; ^2^ Yellowstone to Yukon Conservation Initiative PO Box 733 Sandpoint ID USA; ^3^ U.S. Forest Service Rocky Mountain Research Station Flagstaff AZ USA

**Keywords:** diversity, monitoring, sampling

## Abstract

Winter bait stations are becoming a commonly used technique for multispecies inventory and monitoring but a technical evaluation of their effectiveness is lacking. Bait stations have three components: carcass attractant, remote camera, and hair snare. Our 22,975 km^2^ mountainous study area was stratified with a 5 × 5 km sampling grid centered on northern Idaho and including portions of Washington, Montana, and British Columbia. From 2010–14, we conducted 563 sampling sessions at 497 bait stations in 453 5 × 5 km cells. We evaluated the effectiveness of cameras and hair snare DNA collection at stations to detect species and individual animals, factors affecting DNA viability, the effectiveness of re‐visiting stations, and the influence of elevation, seasonality, and latency on species detections. Cameras were more effective at detecting multiple species than DNA hair snaring. Length of deployment time and elevation increased genetic species ID success but individual ID success rates were increased only by collecting hairs earlier in the season. Re‐visiting stations did not change camera or genetic species detection results but did increase the number of individual genotypes identified. Marten and fisher were detected quickly while bobcat and coyote showed longer latency to detection. Seasonality significantly affected coyote and bobcat detections but not marten, fisher, or weasel. Multispecies bait station study design should incorporate mixed elevation sites with stratified seasonality. Priority should be given to including cameras as components of bait stations over hair snares, unless there is a specific genetic goal to the study. A hair snare component should be added, however, if individual ID or genetic data are necessary. Winter stations should be deployed a minimum of 45–60 days to allow for detection of low density species and species with long latency to detection times. Hair samples should be collected prior to DNA‐degrading late season rain events. Re‐visiting stations does not change which species are detected at stations; therefore, studies with objectives to delineate species presence or distribution will be more effective if they focus on deploying more stations across a broader landscape in lieu of surveying the same site multiple times.

## 1 INTRODUCTION

There is an increasing need for survey techniques to efficiently detect multiple species in single field efforts in order to provide information on species distribution and population status (Boutin, Haughland, Schieck, Herbers, & Bayne, 2009; Butchart et al., 2010; Manley, Zielinski, Schlesinger, & Mori, 2004; Turner et al., 2015). However, species differ in spatial distribution, habitat use, and behavior which can affect detection probabilities for different species when surveyed using a single technique. Multiple species frameworks, therefore, require more complicated technical evaluations than single species techniques (Carvalho, Gonçalves, Guisan, & Honrado, 2015), and multispecies survey techniques should be tested before widespread use is adopted.

Bait stations were used as a research tool for wolverine (*Gulo gulo*) (Copeland, 1993) and other mustelids (Zielinski & Kucera, 1995) beginning in the 1990s. Improvements in DNA and remote camera technology have enabled the use of bait stations to collect wolverine demographic data (Magoun, Long, et al., 2011; Magoun, Valkenburg, Pedersen, Long, & Lowell, 2011) and led to a recent spike in use of this technique primarily for wolverine surveys (e.g., Bradbury & Fisher, 2005; Clevenger, Dorsey, & Heim, 2011; Heinemeyer & Squires, 2014; Kortello & Hausleitner, 2014; Moriarty et al., 2009; Royle, Magoun, Gardner, Valkenburg, & Lowell, 2011) and more recently for multispecies carnivore surveys (Fisher & Bradbury, 2014; SCCMC, 2014).

**Figure 1:**
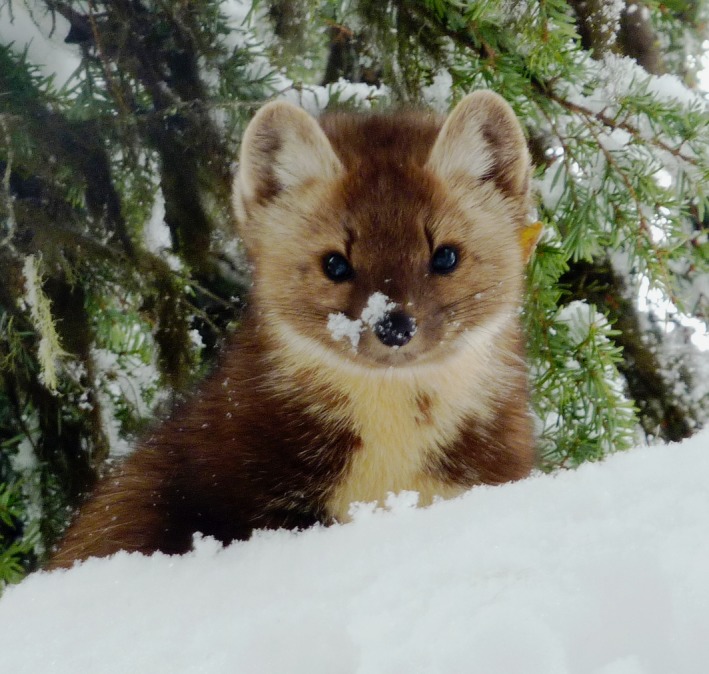
American marten (*Martes americana*). Photograph credit L. Robinson

The methodology of a bait station's separate components is fairly well understood as remote cameras (Burton et al., 2015) and hair snare DNA analysis (e.g., Fisher & Bradbury, 2014; Royle et al., 2011) have been widely tested and implemented independently as species detection tools. However, neither has been extensively evaluated in the context of the bait station method. Fisher and Bradbury (2014) used a relatively small number of bait stations (*n *=* *66) to demonstrate cameras are more efficient than hair snares at detecting fisher (*Pekania pennanti*), marten (*Martes americana*, Figure 1), and wolverine. But despite growing popularity, the performance of the bait station method, either as a single or multispecies technique, remains largely unexplored.

The effects of differences in basic ecology and distribution among species on the performance of the bait station method also need to be evaluated. For example, species often spatially segregate across the landscape by vegetation types and elevation (Witczuk, Pagacz, Gliwicz, & Mills, 2015), possibly as a result of seasonal variables (Krohn, Zielinski, & Boone, 1997). Additionally, species can have different degrees of attraction to bait or latency to detection, which may depend on behavior or density (Fisher & Bradbury, 2014). When using multispecies methods, potential differences in detection probabilities should be considered on a per species basis in study design and the interpretation of results as they can affect occupancy or demographic estimates and modeling outputs.

Our objective was to evaluate the ability of the bait station method to effectively detect species and individual animals within a multispecies framework. During the winters of 2010–2014, we deployed 497 bait stations on a 5 × 5 km grid across the Idaho Panhandle and adjoining mountain ranges. Our goals were to 1) evaluate the effectiveness of remote cameras and hair snaring in detecting multiple species, 2) evaluate the effect of environmental exposure on DNA amplification success rates, 3) evaluate differences in elevation, seasonality, and latency to detection for different species, and 4) evaluate the effectiveness of station revisits in improving species detections of marten and fishers and detection of additional individual fishers.

## 2 METHODS

### 2.1 Study area

**Figure 2:**
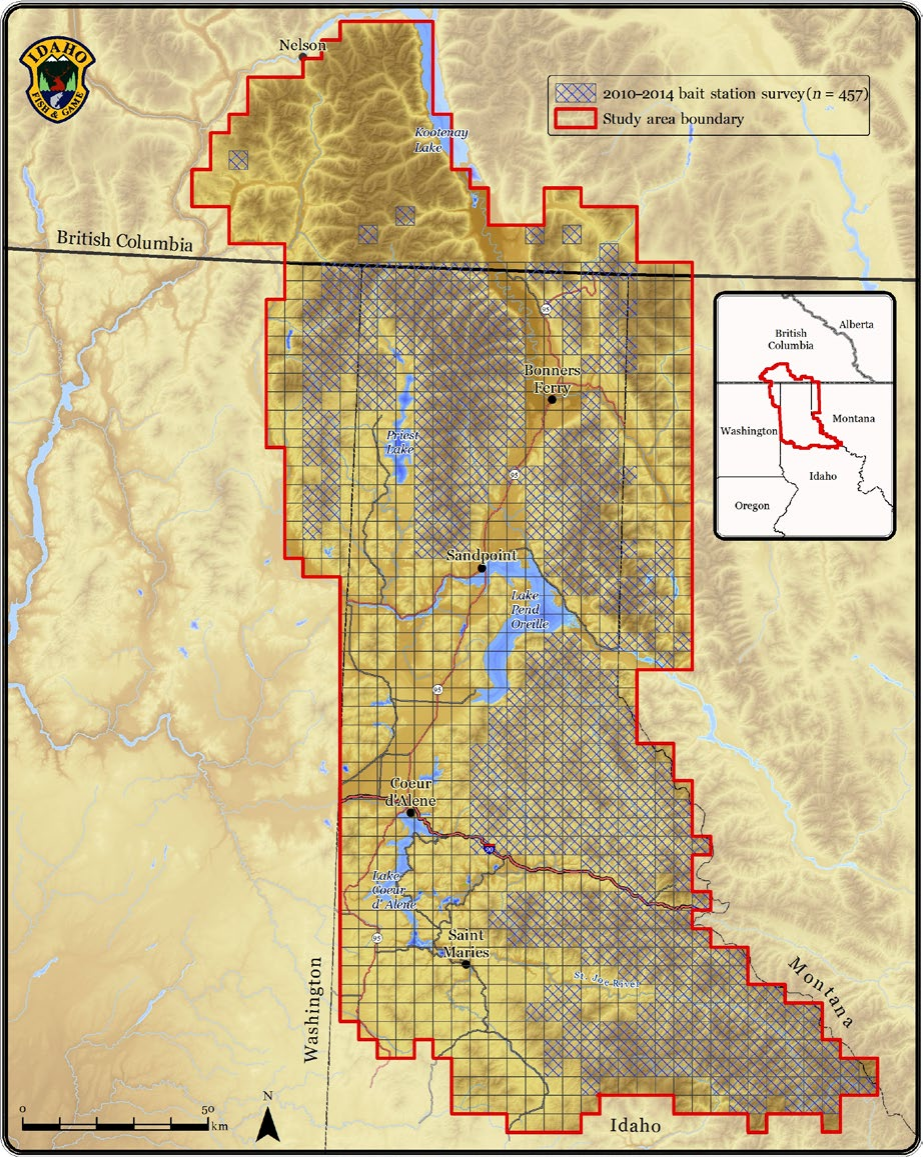
5 km × 5 km grid where carnivore bait stations were deployed during the winters of 2010–2015. Shading indicates the 453 cells surveyed

The study area consisted of a 22,975 km^2^ area centered on the Idaho Panhandle and containing portions of northeastern Washington, northwestern Montana, and southern British Columbia (Figure 2). It comprises portions of the Selkirk, Purcell, West Cabinet, Coeur d' Alene, and Saint Joe mountain ranges. The topography is mountainous, ranging in elevation from 702 to 2326 m. The climate is characterized by mild summers and wet and moderately cold winters. The area is heavily forested and dominated by a diverse mix of conifer species.

### 2.2 Field methods

We stratified the five mountain ranges in our study area with 453 5 × 5 km survey cells (Figure 2) and deployed at least one bait station per cell (Lucid et al. 2016). Topographic features carnivores use for travel, such as saddles, ridges, and heads of drainages (Halfpenny, Thompson, Morse, Holden, & Rezendes, 1995), were prioritized and field personnel deployed stations within 200 m of the assigned location. When re‐visiting stations, field personnel collected hair samples, downloaded pictures, replaced camera batteries, and replaced bait if needed.

### 2.3 Bait station components

#### 2.3.1 Bait tree

**Figure 3:**
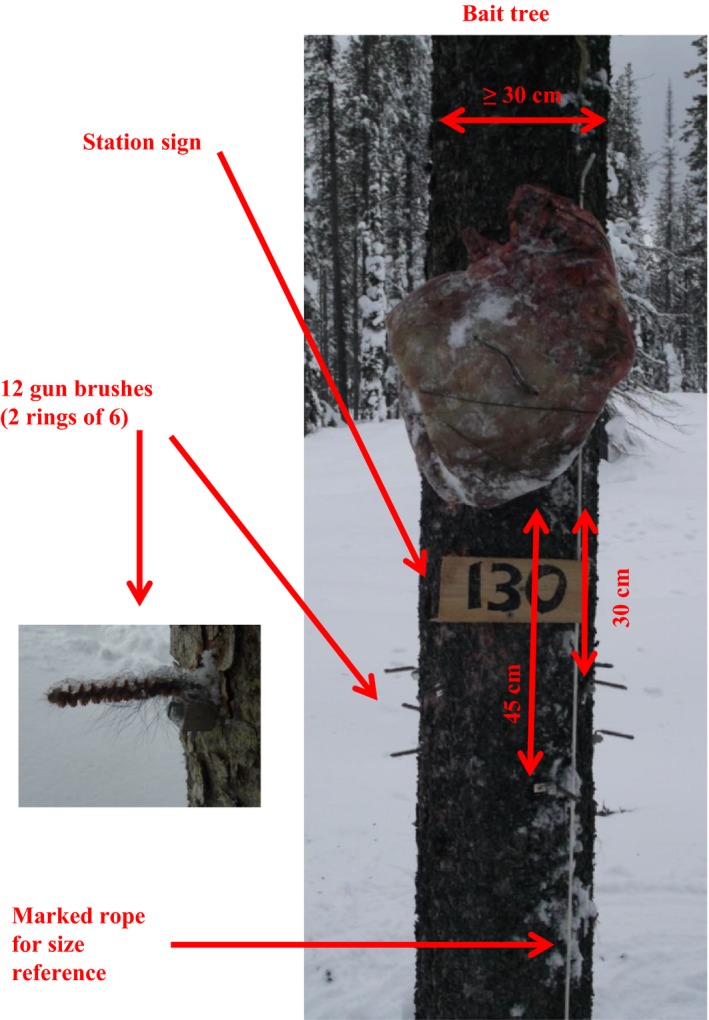
Details of bait tree used in bait station to survey forest carnivores in north Idaho and surrounding drainages during the winters of 2010–2014

**Figure 4:**
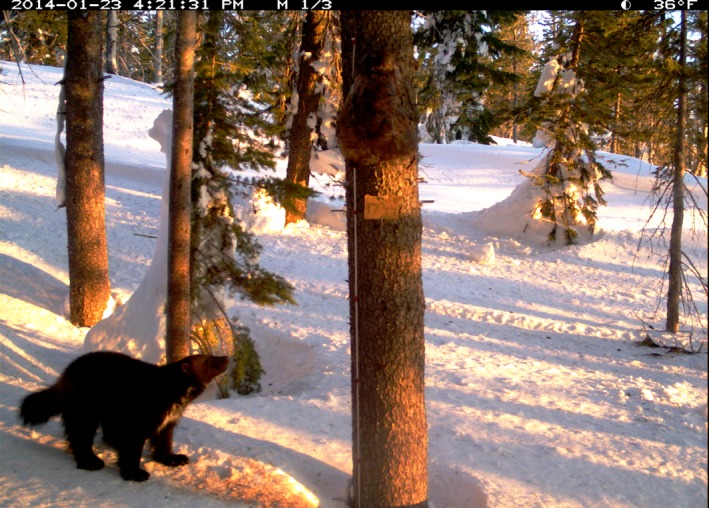
Wolverine (*Gulo gulo*) approaching bait station

We selected live bait trees >30 cm in diameter which were isolated from other trees by at least 1.5 m. This forced the animal to climb the tree from the bottom (and thus contact the hair snare) by removing the option for the animal to jump to the bait from neighboring trees and thereby access the bait from above. We used annealed wire to attach a skinned and frozen beaver carcass or ungulate quarter to the bait tree approximately 2 m above snow level. To ensure bait was firmly attached to the tree, we prewired the frozen bait by drilling holes on either side of the spinal column or leg bone (four holes total). We then tightly wrapped annealed wire around the bone to ensure carnivores would not be able to remove the bait for caching or consumption elsewhere. Rather, carnivores were forced to consume the bait in view of the camera. For a scent lure, we hung a sponge soaked in Gusto (Caven's lures, Minnesota, USA) within 20 m of the bait tree. For a size reference in photographs, we attached a 1.5‐m rope below the bait with reflective tape every 30 cm (Figures 3 and 4).

#### 2.3.2 Hair snare

We used aluminum terminal lugs to affix 12.30 caliber bronze rifle bore gun brushes (henceforth, gun brushes) below the bait in two concentric rings of six at 30 and 45 cm below the bottom of the bait (Figures 3 and 4). To reduce bait contamination of hair samples, we avoided placing gun brushes directly below the bait. Field personnel used latex gloves to remove gun brushes from terminal lugs to avoid contaminating samples with human or pet DNA. Gun brushes with hair were placed in coin envelopes and allowed to dry at room temperature until they were analyzed.

#### 2.3.3 Camera trap

We deployed one remote camera on a tree 2.75–3.25 m from the bait tree. We primarily used Reconyx^™^ cameras (Wisconsin, USA) equipped with ≥ 4GB memory cards and rechargeable NiMH batteries. Cameras were set on high sensitivity to take 3 rapidfire pictures with no delay between triggers; night mode was set to “balanced.” All images collected by remote cameras (*n *=* *722,435) were reviewed independently by two wildlife biologists to identify species.

### 2.4 Genetics methods

We used camera data to inform our selection of hair samples submitted for DNA analysis and only submitted samples from stations which obtained images of fisher, wolverine, lynx, bobcat, or marten (*n *=* *360). We visually inspected hair samples and preferentially submitted samples with the most guard hairs for each station. We submitted an average of 2.8 gun brushes per visit for species ID. Of the samples already identified to species, we then submitted an average of 3.6 per visit for individual genotype.

Samples were analyzed for species and individual identification (ID) at Wildlife Genetics International (WGI; British Columbia, Canada). WGI extracted DNA from hair samples by clipping up to 10 guard hair roots or up to 30 under‐fur hairs to supplement guard hairs. Samples were processed with QIAGEN DNeasy Blood and Tissue Kits (Germany), using QIAGEN's protocol for tissue. The species test was a partial sequence analysis of the mitochondrial 16S rRNA gene. Microsatellite markers were used to determine individual ID by developing genotypes for fisher (seven markers), lynx (11 markers), and wolverine (13 markers).

We used two measures of success in our analyses to assess the efficacy of DNA analysis:

1. Station Success = No of stations producing a species ID or genotype ÷ No of stations analyzed
2. To standardize differing numbers of samples submitted per station we calculated a proportional success rate to use in analyses: Proportional Success  =  # samples successfully amplifying for a station ÷ # samples analyzed for that station.

### 2.5 Statistical analyses

#### 2.5.1 Data subsets

We used our full database to develop four datasets that were used in statistical analyses.

Full Dataset—All image and species ID data included from full deployment length of all stations (*n *=* *497).

Twenty‐eight‐day Truncate—We removed all stations that had a sampling period of < 28 days (*n *=* *19) or experienced a camera error event in the first 28 days of deployment (*n *=* *83). Types of camera errors include camera not triggering for all detections, technical failure of camera or memory card, camera covered by snow or ice, and memory card filled up prematurely. We used first day of camera detection to remove species detected for the first time after 28 days. The resulting dataset consisted of stations which had full image data for the first 28 days of deployment only (*n *=* *395).

Multiple Visit—Only stations that were visited multiple times: *n *=* *54 for second visits, *n *=* *12 for third visits.

DNA Exposure‐ Species ID: Sampling sessions where ≥1 sample was submitted for DNA analysis. Repeat station visits were counted as independent sampling events (*n *=* *360).

Individual ID: Sampling sessions where ≥1 fisher, wolverine, or lynx sample was submitted for individual genotyping (*n *=* *84).

Fisher Genotype: Sampling sessions where ≥1 sample was submitted for fisher genotyping (*n *=* *63).

#### 2.5.2 Elevation

We used the Full Dataset to tally the number of species detected by camera and DNA and calculate mean detection elevations for 11 species.

#### 2.5.3 DNA exposure

We looked at the combined effects on DNA success of the following predictor variables: Number of days the station was deployed (DAYS), date the station was deployed (START), date the station was retrieved (END), and elevation of the station (ELEV). In three sets of analyses, we used proportional success by visit as the response variable for species ID, individual ID, and fisher individual ID. For each response variable, we first ran a correlation analysis to determine whether the predictor variables were highly correlated. None had correlation values > |0.7| so all were retained (Appendices1–1, 1–2, 1–3). We then ran an all‐subsets logistic regression using the Dredge function in R (R Development Core Team 2012) and produced a model averaged result for all models within four AIC units of the best model.

We averaged the AIC weight of these four variables in all models within four AIC units of the best model to measure variable importance. We measured variable effect size in two ways. First, we calculated the relative magnitude of standardized regression coefficients for each variable. Second, we plotted marginal response curves showing the predicted probability of successful genotyping across the range of each variable while holding the other variables constant at their means.

#### 2.5.4 Station revisits

We used the Multiple Visit dataset to compare detections of marten and fisher across revisits at the same station based on camera images. We used genotyping results to compare the number of individual fishers that were detected across visits to the same station. We used a z‐test for proportions to determine whether the proportion of stations detecting marten or fisher was significantly different between the first visit and all visits combined.

In order to determine whether more frequent collections of hair samples resulted in higher DNA success, we used a z‐test for proportions to compared the Station Success and Proportional Success for both species ID and individual ID for stations that were visited multiple times to those that were not revisited.

In order to determine whether revisiting the station affected the latency to detection for marten and fisher, we selected all the stations where a marten or fisher was detected by camera in both the initial and revisit occasion (*n *=* *26 for marten and *n *=* *10 for fisher). We then calculated the mean of the difference between the time to first photograph in the first and second sessions and used a paired t‐test to evaluate significance of differences.

#### 2.5.5 Latency to detection

We used the Full Dataset to determine the overall time to first detection for 11 species by subtracting the date the species was first observed in camera images from the deployment date. We then used a Tukey Honest Significant Difference (HSD) multiple range test to compare differences in latency to detection for eight species. We used the 28‐Day Truncate dataset to produce detection accumulation curves for marten, fisher, bobcat, and coyote by calculating the proportion of detections cumulatively as a function of the number of days deployed.

#### 2.5.6 Seasonality of detection

We used the 28‐Day Truncate dataset to evaluate differences in seasonality of detections for marten, weasel, fisher, bobcat, and coyote. To determine whether there was a seasonal effect in species detections, we standardized across years by defining the first day of the sampling season. For each station, the deployment date was converted to a number between one and 144. Day one was defined as October 30 because this was the earliest setup date in any survey season. We then tabulated detection or no detection (grouping DNA and camera) for each species, and ran a logistic regression analysis to determine whether species detection rate varied as a function of the start date and the start date squared (i.e., linear and unimodal pattern of change in probability over the sampling period). To further investigate possible relationships, we produced Lowess spline plots between detection and start date for bobcat and coyote.

## 3 RESULTS

### 3.1 Species identifiction

Stations were deployed for a median of 39 days (range 12–162 days) with a median deployment date of January 25 (earliest was October 30) and takedown of March 14 (latest was June 30). Most stations (*n *=* *443) were visited once, and 54 stations were revisited one to three times.

Remote cameras detected 28 species (or genera) and DNA analysis detected 14 species (Table 1). The 14 camera‐exclusive species tended not to climb the bait tree (ungulates, canids, and birds) or were relatively small (e.g., mice) (Table 1). Mean detection elevations ranged from 1148 m (bobcat) to 1747 m (lynx) with five species below and six species higher than the mean station elevation (1323 m) (Table 1).

**Table 1:** Species and genera detected by remote camera and DNA analysis at forest carnivore bait stations in the Idaho Panhandle and surrounding drainages during the winters of 2010–2014

Species	Latin name	Detected By			DNA total	Camera total	Stations detecting species by either camera or DNA (% of total stations surveyed)	Median days to first detection^a^	Mean elevation of species detected (m)
		Camera only	DNA only	Camera and DNA					
Marten	*Martes americana*	49	10	207	217	256	266 (54)	7.0	1467
Red squirrel	*Tamiasciurus hudsonicus*	193	1	4	5	197	198 (40)	19.0	1342
Flying squirrel	*Glaucomys sabrinus*	183	1	9	10	192	193 (39)	13.0	1189
Weasel	*Mustela spp*.	108	2	4	6	112	114 (23)	10.5	1209
Gray jay^b^	*Perisoreus canadensis*	74	0	0	0	74	74 (15)	12.5	1410
Snowshoe hare^b^	*Lepus americanus*	70	0	0	0	70	70 (14)		
Steller's jay^b^	*Cyanocitta stelleri*	62	0	0	0	62	62 (12)	15.5	1450
Fisher	*Pekania pennanti*	9	1	48	49	57	58 (12)	12.0	1289
Moose^b^	*Alces alces shirasi*	55	0	0	0	55	55 (11)		
Coyote^b^	*Canis latrans*	50	0	0	0	50	50 (10)	27.0	1267
Bobcat	*Lynx rufus*	21	2	27	29	48	50 (10)	19.0	1148
White‐tailed deer^b^	*Odocoileus virginianus*	37	0	0	0	37	37 (7)		
Elk^b^	*Cervus elaphus*	33	0	0	0	33	33 (7)		
Raven^b^	*Corvus corax*	23	0	0	0	23	23 (5)		
Wolf^b^	*Canis lupus*	16	0	0	0	16	16 (3)		
Black bear	*Ursus americanus*	14	1	1	2	15	16 (3)		
Clark's nutcracker^b^	*Nucifraga columbiana*	13	0	0	0	13	13 (3)		
Mouse	*Peromyscus spp*.	10	0	0	0	10	10 (2)		
Mink	*Mustela vison*	2	0	7	7	9	9 (2)		
Raptor^b^	*Accipitridrae*	9	0	0	0	9	9 (2)		
Wolverine	*Gulo gulo*	2	0	6	6	8	8 (2)	29.0	1567
Red Fox	*Vulpes vulpes*	4	0	2	2	6	6 (1)		
Mule deer^b^	*Odocoileus hemionus*	5	0	0	0	5	5 (1)		
Raccoon	*Procyon lotor*	2	0	1	1	3	3 (1)		
Cougar^b^	*Puma concolor*	3	0	0	0	3	3 (1)		
Lynx	*Lynx canadensis*	0	0	2	2	2	2 (0)	43.0	1747
Grizzly bear	*Ursus arctos*	1	0	1	1	2	2 (0)		
Striped skunk	*Mephitis mephitis*	0	0	1	1	1	1 (0)		

a Days to first detection were calculated by subtracting the first date the species is identified on camera minus the date of station setup.

b Indicates species that did not climb the bait tree and therefore did not leave a DNA sample.

#### 3.1.1 DNA success rates

We collected 3,945 DNA samples and submitted 1,011 for species ID (Table 2).

**Table 2:** DNA success rates for species ID and genotyping analyses performed on samples collected at forest carnivore bait stations in the Idaho Panhandle and adjoining mountain ranges during the winters of 2010–2014

Year	No. of stations surveyed	Mean No of days deployed	No. of stations revisited	Mean No of days between revisits	Total No of sampling sessions	Sampling sessions with ≥1 sample analyzed for species ID	Proportional species ID success	Sampling sessions with ≥1 sample analyzed for individual ID	Proportional genotype success	Sampling sessions with ≥1 sample analyzed for fisher genotype	Proportional fisher genotype success	Total No of samples collected	Samples analyzed for species ID	Samples producing species ID	Samples analyzed for genotype	Samples producing genotype
2010	16	89	16	44	32	31	0.81	15	0.63	1	1	124	124	104	21	10
2011	17	34	12	18	29	24	0.91	16	0.70	15	0.68	337	184	161	132	86
2012	86	54	24	29	122	88	0.95	30	0.76	28	0.76	1037	216	201	80	61
2013	97	45	2	28	99	72	0.89	6	0.61	5	0.53	703	139	125	13	7
2014	281	46	0		281	147	0.90	17	0.52	13	0.61	1744	348	310	56	37
Total	497		54		563	360		84		62		3945	1011	901	302	201

Species was successfully identified for 901 of 1,011 samples analyzed (89%). Of 302 samples analyzed for individual ID, 201 (67%) successfully produced a genotype (Table 2). Species ID was successful significantly more frequently than individual ID (Z = 9.1371, *p *< .0001). Because some stations included multiple visits, we used the results of each sampling session to evaluate Station Success. We submitted at least one sample for species ID from 319 total stations comprising 360 sampling sessions. Of those, 345 sessions (96%) successfully produced a species ID. We submitted at least one sample for individual ID from 84 sampling sessions. Of those, 69 (82%) successfully produced a genotype.

### 3.2 DNA exposure

#### 3.2.1 Species ID

**Figure 5:**
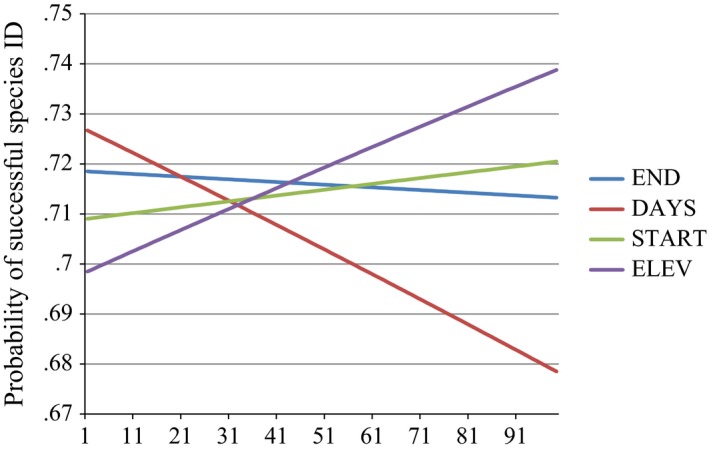
Marginal response curve plot showing the probability of successful species ID (*n *=* *360) for each variable across its range, holding all other variables constant at their medians

Based on AIC variable importance, ELEV was the most important variable and occurred in all models within four AIC units of the best individual model. DAYS was the second most important variable, present in 84% of models within four AIC units of the top model followed by END and START with 48% and 32% involvement in models within four AIC units of the best model, respectively. Based on magnitude of standardized regression coefficients, DAYS was the most influential variable, followed by ELEV which was 77.8% as influential as DAYS, followed by END and START, which were 25.7% and 17.5% as influential as DAYS, respectively (Table 3). The marginal response curve plot shows that DAYS is the most influential variable, with a change from 72.6% successful species ID at the lowest deployment length, decreasing to 67.8% success at the maximum number of collected samples (Figure 5). ELEV is the second most influential variable, with successful species ID increasing from 69.8% at the lowest elevation to 73.9% at the highest elevation. START and END have relatively little effect on species ID with total change in probability of successful species ID of around 1% across the range of these variables.

**Table 3:** Relative magnitude of standardized regression coefficients for species ID

Variable	Estimate of coefficient	Relative magnitude
DAYS	−0.03946	1
ELEV	0.0307	0.778003
END	0.01016	0.257476
START	−0.00689	0.174607

#### 3.2.2 Individual genotype (Wolverine, Lynx, and Fisher)

**Figure 6:**
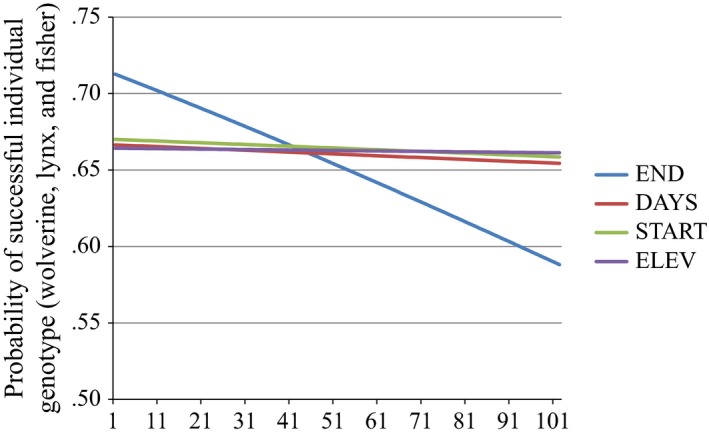
Marginal response curve plot showing the probability of successful individual genotype of wolverine, lynx, and fisher (*n *=* *84) for each variable across its range, holding all other variables constant at their medians

Based on AIC results, END was the most important variable, and occurred in 85% of models within 4 ΔAIC units of the best overall model. DAYS, START, and ELEV were included in 45%, 32%, and 30% of models within four AIC units, respectively. Based on magnitude of standardized regression coefficients, END was the most influential variable, followed by DAYS, ELEV, and START (25.2%, 10.8% and 9.4%, respectively; Table 4). The marginal response curve plot shows that END is the most influential variable, with a change from 71% successful species at the earliest end date, decreasing to 58.8% success at the maximum end date (Figure 6). The other variables had a total effect of <1% change in probability of successful individual ID across the ranges of those variables.

**Table 4:** Relative magnitude of standardized regression coefficients for individual genotype (wolverine, lynx, and fisher)

Variable	Estimate of coefficient	Relative magnitude
END	−0.10397	1
DAYS	−0.02621	0.252046
ELEV	−0.01128	0.108475
START	−0.00979	0.094192

#### 3.2.3 Individual fisher genotype

**Figure 7:**
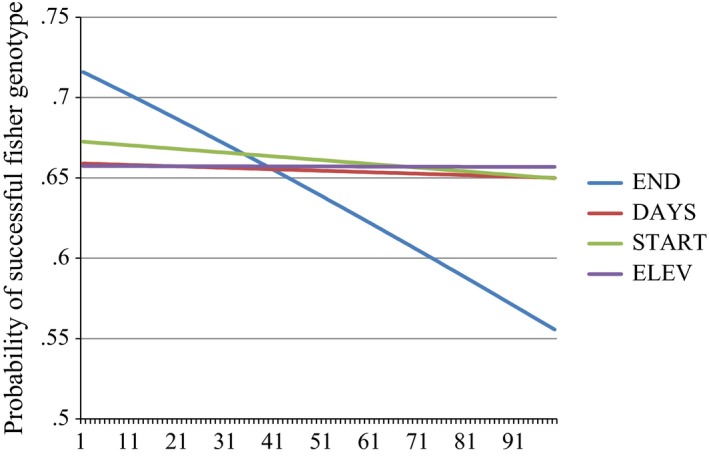
Marginal response curve plot showing the probability of successful fisher genotype (*n *=* *63) for each variable across its range, holding all other variables constant at their medians

Based on AIC results, END was the most important variable and was included in 87% of models within four ΔAIC units of the best overall model. DAYS, START, and ELEV occurred in 40%, 27%, and 11% of models within four AIC units, respectively. Based on magnitude of standardized regression coefficients, END was by far the most influential variable in terms of effect size (Table 5). The next largest effect size was for START, which was 17.5% as influential as END, followed by DAYS which was 7.3% as influential as END. ELEV was the least impactful variable, with 0.5% of the influence of END on genotyping proportion success. The marginal response curve plot shows that END is vastly more important than any other variable, with a change in probability of successful genotyping from 71.5% at the earliest pickup date to 55.3% at the latest pickup date (Figure 7).

**Table 5:** Relative magnitude of standardized regression coefficients for fisher genotyping

Variable	Estimate of coefficient	Relative magnitude
END	−0.13372	1
START	−0.02343	0.175229
DAYS	−0.00973	0.072796
ELEV	−0.00072	0.005382

### 3.3 Station revisits

We revisited 54 stations one to three times for a total of 120 sampling sessions (Table 2). Mean deployment length of revisited stations was 25 days for the first sampling period (*n *=* *54), 36 days for the second sampling period (*n *=* *54), and 29 days for the third sampling period (*n *=* *12).

#### 3.3.1 Species detection: marten

Of the 54 stations revisited at least once, 38 detected marten either by remote camera or DNA during the first sampling session. During the second session, marten were redetected at 34 of the 38 stations (89%) where they were detected during the first session and at an additional two new stations that had not detected marten during the first session. Ten of 12 stations that were visited three times detected marten on the first or second visit. During the third visit, marten were re‐detected at eight (80%) stations and were not detected at the two stations which had not previously detected them. There was not a significant difference between the proportion of stations detecting marten on the first visit and the proportion of stations detecting marten across all revisits (Z = 0.396, *p *=* *.689).

#### 3.3.2 Species detection: fisher

Fifteen of 54 stations detected fisher during the first sampling session. During the second visit, fisher were redetected at 12 (80%) stations and were detected at three new stations. Six of 12 stations that were visited three times detected fisher on the first or second visit. During the third visit, fisher were re‐detected at four (67%) stations and were not detected at any new stations. There was not a significant difference between the proportion of stations detecting fisher on the first visit and the proportion of stations detecting fisher across all revisits (Z = 0.617, *p *=* *.535).

#### 3.3.3 Individual detection: fisher

Using genotyping results, 13 fishers were detected at 15 stations during the first sampling session. During the second session, six of the original 13 fishers were re‐detected and 10 new individual fishers were detected. During the third visit, three individual fishers were detected at 12 stations. All three individuals had been previously detected, although one was detected at a new station. There was a statistically significant difference in the number of individual fishers detected on the first visit vs. all subsequent visits to the same station (t‐test, *p *=* *3.727e‐05).

#### 3.3.4 DNA success

In stations with at least one sample submitted for species ID analysis, 48 of 49 (98%) re‐visited stations and 256 of 270 (95%) nonrevisited stations successfully produced a species ID. This difference is not statistically significant (Z = 0.9281, *p *=* *.352). Proportional species ID success was 0.89 for revisited stations compared with 0.91 for stations that were not revisited, which was also not statistically significant. In stations with at least one sample submitted for individual ID analysis, 91% of revisited (*n *=* *32) and 76% of nonrevisited (*n *=* *37) stations successfully produced an individual ID, which was not a statistically significant difference (Z = 1.6525, *p* = .0989). Proportional individual ID success was 0.74 for revisited stations compared with 0.62 for stations that were not revisited, which was also not a statistically significant difference.

#### 3.3.5 Latency to detection

Both marten and fisher showed a marked decrease in the number of days to first detection during the second sampling session. For stations where a marten was detected by camera in both the first and second sampling sessions, date of first detection at the same station decreased by an average of 8.35 days (*p *=* *.0003). Mean detection time decreased by 3.70 days for stations where fishers were detected by camera in both the first and second sampling periods (*n *=* *10) meaning that fisher on average revisit a re‐baited station nearly 4 days sooner than they originally visited the same station on the initial sampling period. The difference was not significant at the 0.05 level (*p *=* *.081) in part due to relatively small sample size of stations detecting fisher in both initial and revisit sessions.

### 3.4 Latency to detection

Median time to first detection for 11 species ranged from 7.0 days to 43.0 days (Table 5). Marten and fisher responded relatively quickly to the bait station stimulus. Median time to first detection was 7.0 days (*n *=* *248 stations) for marten and 12.0 days (*n *=* *50 stations) for fisher (Table 1). Tukey's HSD multiple range tests of differences between pairs of species in mean time to first detection found that coyote was significantly longer to first detection than all other species except bobcat (*p *=* *.9579) and wolverine (*p *=* *.7893; Table 6). Marten was significantly faster to first detection than all other species except fisher (*p *=* *.1044), weasel (*p *=* *.7553), and wolverine (*p *=* *.3213).

**Table 6:** Tukey's Honest Significant Difference multiple range tests for difference between eight species in time to first detection across stations

Pair	Estimate	Standard Error	*t*	Pr(>|*t*|)
Coyote–bobcat	6.9310	6.5936	1.051	.9579
Fisher–bobcat	−11.4524	6.3691	−1.798	.5770
Flying squirrel–bobcat	−8.1047	6.1029	−1.328	.8659
Marten–bobcat	−18.8907	5.9312	−3.185	.0262
Red squirrel–bobcat	−7.6814	6.0405	−1.272	.8902
Weasel–bobcat	−14.8261	6.3246	−2.344	.2352
Wolverine–bobcat	−4.000	8.9167	−0.449	.9998
Fisher–coyote	−18.3834	3.9863	−4.612	<.001
Flying squirrel–coyote	−15.0357	3.5454	−4.241	<.001
Marten–coyote	−25.8217	3.2409	−7.967	<.001
Red squirrel–coyote	−14.6125	3.4369	−4.252	<.001
Weasel–coyote	−21.7571	3.9149	−5.558	<.001
Wolverine–coyote	−10.931	7.4050	−1.476	.7893
Flying squirrel–fisher	3.3477	3.1081	1.077	.952
Marten–fisher	−7.4383	2.7557	−2.699	.1044
Red squirrel–fisher	3.771	2.9838	1.264	.8934
Weasel–fisher	−3.3737	3.5237	−0.957	.9748
Wolverine–fisher	7.4524	7.2058	1.034	.9614
Marten–flying squirrel	−10.786	2.0672	−5.218	<.001
Red squirrel–flying squirrel	0.4232	2.3627	0.179	1.000
Weasel–flying squirrel	−6.7214	3.0159	−2.229	.2952
Wolverine–flying squirrel	4.1047	6.9716	0.589	.9987
Red squirrel–marten	11.2093	1.8751	5.978	<.001
Weasel–marten	4.0646	2.6514	1.533	.7553
Wolverine–marten	14.8907	6.8218	2.183	.3213
Weasel–red squirrel	−7.1447	2.8876	−2.474	.1774
Wolverine–red squirrel	3.6814	6.9171	0.532	.9993
Wolverine–weasel	10.8261	7.1666	1.511	.7690

**Figure 8:**
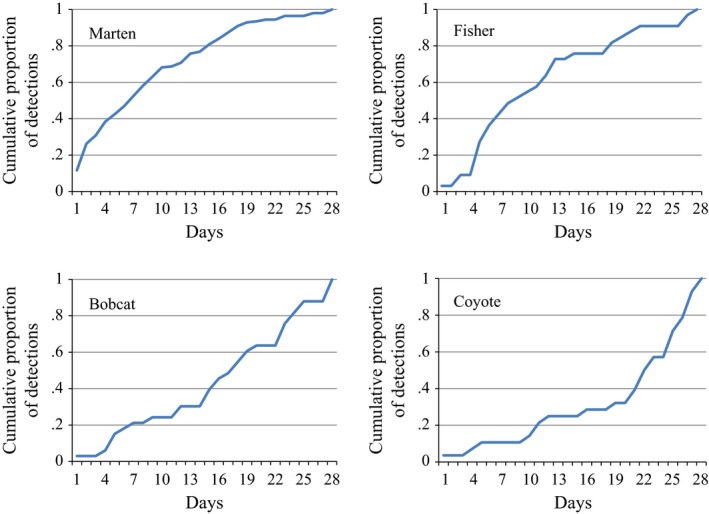
Detection accumulation curves based on a 28‐day sampling period of remote camera images obtained at forest carnivore bait stations in the Idaho Panhandle and adjacent mountain ranges during the winters 2010–2014

**Figure 9:**
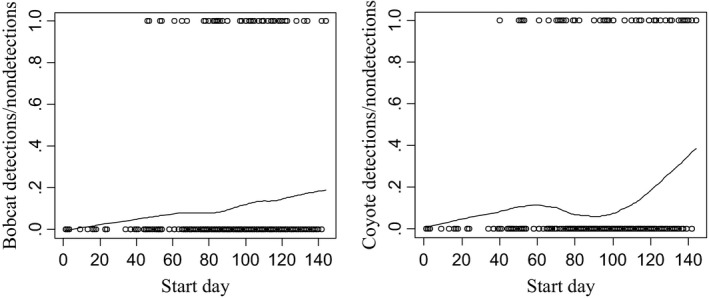
Lowess spline plots of detections for bobcat (left) and coyote (right) based on a 28‐day sampling period of remote camera images obtained from forest carnivore bait stations in the Idaho Panhandle during the winters of 2010–2014

Detection accumulation curves showed 70% of marten detections occurred in the first 10 days of station deployment. Similarly, by day 13, >70% of fisher detections had occurred (Figure 9). Bobcat and coyote showed a substantially slower reaction to bait stations (Table 1). Median time to first detection was 19.0 days (*n *=* *48 stations) for bobcats and 27.0 days (*n *=* *51 stations) for coyotes (Table 1). For bobcat, detection accumulation curves showed avoidance for the first three days after which, the curve rises steadily to 62% of first detections having occurred by day 21. Coyotes had the longest latency to detection with only 25% of first detections occurring by day 15, with the curve rising steeply after day 20 (Figure 8).

### 3.5 Seasonality of detection

There were no significant linear (*p *=* *.835) or quadratic (*p *=* *.541) relationships between date of deployment and marten detection. Nor was there a significant relationship between deployment date and probability of detection of weasel in either linear (*p *=* *.193) or quadratic (*p *=* *.221) form of relationship or fishers (linear *p *=* *.077, quadratic *p *=* *.0697), indicating that marten, weasel, and fisher detection probabilities do not vary across the seasonal period sampled.

There was a significant relationship between detection probability and deployment date in both the linear and quadratic forms of the relationship for both bobcats (linear *p *=* *.0049, quadratic *p *=* *.0050) and coyotes (linear *p *=* *.0012, quadratic *p *=* *.00013). The Lowess plot for bobcat shows a general increase in probability of detection throughout the sampling period, indicating higher probability of detecting bobcat with the bait station method in late winter and early spring than in fall or early winter (Figure 9). The Lowess plot for coyote indicates a large increase in detection probability for coyote during the last 30 days of the sampling period, and a midseason lull between days 70 and 110 where detection probability is low (Figure 9).

## 4 DISCUSSION

We examined the efficiency of the bait station method as a multispecies survey technique on a multi and per species basis. Remote cameras detected 28 species of carnivores, ungulates, and birds at bait stations, and species‐specific detection was affected by various factors which multispecies studies should account for.

### 4.1 Species identifiction

Our results corroborate other studies (e.g., Fisher & Bradbury, 2014) that detected more species with cameras than DNA. Genetic species ID is limited by 1) preclusion of nontree climbing species and 2) our ID test being limited to one species per gun brush. This limitation may allow species with short latencies to detection (e.g., marten) to “fill up” gun brushes, thereby causing DNA masking. This would bias DNA results against low density species such as wolverine and lynx or species, such as bobcat, with long response times. Even if genetic techniques evolve to address this issue, we would still strongly recommend against the use of bait stations with hair snares alone to avoid this bias in addition to allowing for detection of nontree climbing species.

### 4.2 DNA exposure

Researchers often limit hair sample exposure time (e.g., Kendall & McKelvey, 2008) in an effort to reduce the negative effects of solar radiation and moisture on DNA integrity (Dumond, Boulanger, & Paetkau, 2015; Stetz, Seitz, & Sawaya, 2014). We expected our dense forest canopy to prevent solar caused DNA degradation (Kendall & McKelvey, 2008), but the frequent late season rain events in our study area presented an opportunity to investigate the effects of moisture on DNA integrity over long winter deployments. We found days deployed to be less statistically important than elevation in species ID success rates. Because DNA exposed to freezing winter conditions is more likely to produce successful genotypes than DNA exposed to wet spring conditions (Maudet, Luikart, Dubray, Von Hardenberg, & Taberlet, 2004; Oehm, Juen, Nagiller, Neuhauser, & Traugott, 2011), we interpret our results to mean higher elevation stations had more successful species ID because they were more likely to experience fewer DNA degrading rain events. However, given the relatively large sample size, the effect of these two variables on proportional species ID success was marginal. We saw a < 0.05 unit change in the response variable over the range of values for both elevation and days deployed, indicating species ID is robust to long winter deployments despite hair samples being on unprotected gun brushes.

Individual genotyping requires a greater quantity and quality of DNA and is more to sensitive to exposure than species ID. However, end date was the only predictor variable that significantly influenced genotyping success, with samples collected later in the season less likely to produce genotypes. This suggests late season rain events are the primary factor influencing the modest decline in genotyping success (0.71–0.59 over our range of end dates).

Our results indicate exposed bait station hair samples will retain adequate DNA over long winter deployments as long as they are collected before spring rain events. This is an important advantage to winter season surveys as stations with long deployments will increase probability of detecting low‐density species.

### 4.3 Station revisits

Mustelids exhibit strong positive responses to baited traps (Hamm, Diller, Klug, & McDonald, 2003; Mowat & Paetkau, 2002; Royle et al., 2011) suggesting that studies focused on species detection/nondetection would revisit baited stations primarily to obtain additional new species detections. However, similar to Fisher and Bradbury (2014), we found revisits did not significantly increase the number of stations detecting marten or fisher by camera or DNA. We did, however, find a substantial increase in the number of individual fishers detected at stations across revisits.

Longer deployments may increase the likelihood of additional individuals visiting a station as a “scavenger footprint” of scent trails and meat caches develops at the survey site (R. Yates, personal communication). However, without gun brush replacement, new individuals may not be detected genetically because brushes are already filled up. The increase in detection of individual fishers when gun brushes are replaced indicates more individuals are visiting stations than are identified in photographs and suggests the deployment of clean gunbrushes during revisits mitigates DNA masking effects.

While revisits appear to be of some value when study objectives include collecting genetic material from multiple individual animals, our results show the odds of a station's response (detection or nondetection) changing upon revisit are low.

### 4.4 Species differences: elevation, seasonality, and latency,

As we expected, we detected strong patterns of species stratification across elevational gradients (e.g., Ruggiero, Aubry, Buskirk, Lyon, & Zielinski, 1994). We found a clear pattern of marten, wolverine, and lynx being associated with higher elevations, and coyote, fisher, and bobcat with lower elevations.

In addition, because rapidly changing snowpacks may influence animal movement (Dowd, Gese, & Aubry, 2014; Krohn et al., 1997) and logistical challenges of remote winter field surveys make it unlikely surveys will be completed within the same portion of a winter season (Royle et al., 2011), it is important to consider seasonality on a per species basis during study design. Ubiquitous marten and weasel showed no seasonal differences in detection rates likely because they had continuous access to the surveyed area. Conversely, bobcat and coyote had a positive association with low elevation sites and showed a marked increase in detection as the season progressed. This could be related to the relatively high foot loading of these species (Zielinski, 2014), resulting in increased access to bait station sites as snow melted.

Similar to other survey methods (Long, Donovan, Mackay, Zielinski, & Buzas, 2007; Zielinski, Truex, Ogan, & Busse, 1997), we found a relatively short latency to first detection for marten (7 days) and fisher (12 days) to bait stations. This indicates bait stations can be reliably used to rapidly detect these two species when present. Bait stations detect wolverine and lynx more effectively in areas of high (Clevenger et al., 2011; Kortello & Hausleitner, 2014; SCCM, 2014) than low (Moriarty et al., 2009) density. We conclude our latency to detection was longer than latency rates inferred from other studies (Clevenger et al., 2011; Kortello & Hausleitner, 2014; Southwestern Crown Carnivore Monitoring Team, 2014) because we have relatively few lynx and wolverine in our study area. Our small wolverine and lynx sample was gained through densely spaced stations across a large landscape for relatively long deployment lengths.

## 5 CONCLUSIONS

Bait station study design should incorporate mixed elevation sites with stratified seasonality to capture a more accurate representation of species assemblages. We recommend always incorporating remote cameras into bait stations for the purpose of species ID and only adding a hair snare component if individual ID or genetic data are necessary for analyses such as population connectivity (e.g., Cushman et al. 2006; Wasserman et al. 2010) or demography (e.g., McCall 2009). Although common species were detected quickly, we recommend deploying bait stations for a minimum of 45–60 days of a winter season to allow for detection of species which may occur at low densities or have long latency to detection times. If hair samples are to be collected, DNA will maintain integrity for this amount of time or longer but hair samples should be collected prior to DNA degrading late season rain events. Re‐visiting stations does not affect which species are detected. Therefore, we recommend studies with objectives to delineate species presence or distribution will be more effective if they focus on deploying more stations across a broader landscape in lieu of surveying the same site multiple times. By applying these recommendations, researchers and managers can use winter bait stations as an effective tool for multispecies inventory and monitoring.

## CONFLICT OF INTEREST

None declared.
