# Supplementary material for: Winter bait stations as a multispecies survey tool
Source: Ecol Evol. 2017 Jul 27;7(17):6826–38. doi: 10.1002/ece3.3158 (PMC5587484; doi:10.1002/ece3.3158)
Supplement: Supplementary file 1 [file ECE3-7-6826-s001.docx]

Appendices.

1-1. Correlation analysis results for variables used in analysis of Species ID.

|  | DAYS | START | END | ELEV | PROP |
| --- | --- | --- | --- | --- | --- |
| DAYS | 1 | -0.15447 | 0.619141 | 0.138135 | -0.16935 |
| START | -0.15447 | 1 | 0.680215 | 0.006022 | 0.07082 |
| END | 0.619141 | 0.680215 | 1 | 0.107272 | -0.06935 |
| ELEV | 0.138135 | 0.006022 | 0.107272 | 1 | 0.118769 |
| PROP | -0.16935 | 0.070819 | -0.06935 | 0.118769 | 1 |

1-2. Correlation analysis results for variables used in analysis of individual genotype (lynx, wolverine, fisher).

|  | DAYS | START | END | ELEV | PROP |
| --- | --- | --- | --- | --- | --- |
| DAYS | 1 | -0.36168 | 0.611473 | 0.242377 | -0.18372 |
| START | -0.36168 | 1 | 0.516539 | 0.039329 | -0.19027 |
| END | 0.611473 | 0.516539 | 1 | 0.255988 | -0.33022 |
| ELEV | 0.242377 | 0.039329 | 0.255988 | 1 | -0.15478 |
| PROP | -0.18372 | -0.19027 | -0.33022 | -0.15478 | 1 |

1-3. Correlation analysis results for variables used in analysis of individual fisher genotype.

|  | DAYS | START | END | ELEV | PROP |
| --- | --- | --- | --- | --- | --- |
| DAYS | 1 | -0.54283 | 0.585378 | 0.121563 | -0.15636 |
| START | -0.54283 | 1 | 0.36315 | 0.2014 | -0.30608 |
| END | 0.585378 | 0.36315 | 1 | 0.32929 | -0.46895 |
| ELEV | 0.121563 | 0.2014 | 0.32929 | 1 | -0.22071 |
| PROP | -0.15636 | -0.30608 | -0.46895 | -0.22071 | 1 |

A2. Model averaged results for species ID

| Variable | Estimate | Stderror | Adjusted | Pr(>\|z\|) |
| --- | --- | --- | --- | --- |
| (Intercept) | 8.45E-01 | 6.16E-02 | 13.668 | <2e-16 |
| DAYS | -1.51E-03 | 8.27E-04 | 1.821 | 0.0686 |
| ELEV | 8.88E-05 | 3.61E-05 | 2.448 | 0.0144 |
| END | -1.83E-04 | 7.31E-04 | 0.25 | 0.8027 |
| START | 3.52E-04 | 7.63E-04 | 0.462 | 0.6443 |

A3. Model averaged results for individual genotype (wolverine, lynx, and fisher).

| Variable | Estimate | StdError | Z | Pr(>\|z\|) |
| --- | --- | --- | --- | --- |
| (Intercept) | 1.15E+00 | 1.84E-01 | 6.165 | <2e-16 |
| END | -2.95E-03 | 1.77E-03 | 1.656 | 0.0978 |
| ELEV | -3.10E-05 | 7.92E-05 | 0.387 | 0.6987 |
| DAYS | -8.55E-04 | 1.74E-03 | 0.487 | 0.6262 |
| START | -3.37E-04 | 1.49E-03 | 0.225 | 0.8221 |

A4. Model averaged results for fisher genotype.

| Variable | Estimate | StdError | Z | Pr(>\|z\|) |
| --- | --- | --- | --- | --- |
| (Intercept) | 1.29E+00 | 1.87E-01 | 6.756 | <2e-16 |
| END | -4.02E-03 | 2.12E-03 | 1.878 | 0.0603 |
| DAYS | -2.96E-04 | 1.88E-03 | 0.156 | 0.8757 |
| START | -8.11E-04 | 1.93E-03 | 0.418 | 0.6759 |
| ELEV | -2.30E-06 | 5.25E-05 | 0.043 | 0.9658 |
